# Prevalence and spatiotemporal distribution of African swine fever in Lithuania, 2014–2017

**DOI:** 10.1186/s12985-018-1090-8

**Published:** 2018-11-19

**Authors:** Arnoldas Pautienius, Juozas Grigas, Simona Pileviciene, Ruta Zagrabskaite, Jurate Buitkuviene, Gediminas Pridotkas, Rolandas Stankevicius, Zaneta Streimikyte, Algirdas Salomskas, Dainius Zienius, Arunas Stankevicius

**Affiliations:** 1 Faculty of Veterinary Medicine, Department of Anatomy and Physiology, Lithuanian University of Health Sciences, Tilzes str. 18, Kaunas, Lithuania; 2 National Food and Veterinary Risk Assessment Institute, J. Kairiukscio str. 10, Vilnius, Lithuania; 3 Faculty of Animal Husbandry Technology, Department of Animal Breeding and Nutrition, Lithuanian University of Health Sciences, Tilzes str. 18, Kaunas, Lithuania; 4 Faculty of Veterinary Medicine, Institute of Microbiology and Virology, Lithuanian University of Health Sciences, Tilzes str. 18, Kaunas, Lithuania; 5 Faculty of Veterinary Medicine, Department of Pathobiology, Lithuanian University of Health Sciences, Tilzes str. 18, Kaunas, Lithuania

## Abstract

**Background:**

The emergence in 2014 and persistence of African Swine Fever (ASF) in Lithuania has been linked to infected wild boar movement and close contact with the carcasses of other infected wild boars. Over time the number of reported cases of ASF in wild boars gradually increased, but no detailed epidemiological data has been available. Therefore, the objective of the present study was to determine ASF virus prevalence in wild boars and domestic pigs during the 2014–2017 period and further explore the current geographical distribution of the virus.

**Results:**

Our study results show that ASF virus prevalence in hunted wild boars using PCR analysis increased from 0.83% (95% CI 0.69–0.98) to 2.27% (95% CI 2.05–2.48) from 2014 to 2016 respectively. However, there was a dramatic jump in the number of ASF positive wild boars cases in 2017 resulting in prevalence of 12.39% (95% CI 11.91–12.86) (*p* < 0.05).

The average prevalence of ASF-specific antibodies in wild boar population during years 2014–2017 was 0.45% (95% CI 0.39–0.51) based on ELISA test results.

Prevalence of ASF virus in domestic pigs ranged from 0.24% (95% CI 0.17% - 0.32) in 2015 to 2.74% (95% CI 2.33% - 3.15) in 2017. The average seasonal prevalence of ASF virus in pigs was statistically significant (*p* < 0.05) and ranged from 0% in spring to 3.68% (95% CI 3.32–4.05) in summer. Correlation between the pig density and number of recorded pig ASF cases in affected regions was only found in 2017 (*R* = 0.78, *p* < 0.05). No correlation was detected between the wild boar density and number of recorded pig or wild boar ASF - positive cases.

**Conclusions:**

This study provides the first results of ASF virus prevalence changes in Lithuania during the 2014–2017. The overall results confirm the relatively high prevalence of ASF virus in wild boar that was gradually increasing from 2014 to 2017. In the last year of study, the number of ASF positive cases in both domestic pigs and wild boars had unexpectedly increased several times. A better understanding of current status of the disease will enable better control and prevent further spread of ASF virus in Western Europe.

## Introduction

African swine fever (ASF) is a contagious hemorrhagic viral disease of swine and other hosts belonging to the family *Suidae* that has recently emerged in several European countries. The disease is caused by enveloped double-stranded DNA virus, the single member of the family *Asfarviridae* [1, 2]. African swine fever virus (ASFV) is remarkably infectious and has devastating effect on local and international pork industry, since the mortality rate of ASF in domestics pigs may reach 100% [3–5]. Moreover, indirect economic loss from trade restrictions is also a relevant factor.

ASF was eradicated in most of Europe in 1990. However, in 2007 ASFV was introduced to the continent for the second time due to single incident in Georgia, from where it rapidly spread to neighboring countries [6, 7]. Since then ASFV has been identified in Russia, Armenia, Azerbaijan, Ukraine, Belarus, Lithuania, Latvia, Estonia and Poland [8–12].

Moreover, prior suggestions [13, 14] on the further spread of ASF virus appeared to be true. The latest reports of the ASF includes Czech Republic, Moldova, Romania, Hungary and Belgium [15–19] that in turn proves ASFV to be an incessant tremendous threat for the rest of Europe.

The virus is very resistant in tissues and the environment, contributing to its transmission over long distances. ASFV is transmitted by variety of mechanisms, including direct contact between infected pigs or by contact with infectious secretions or excretions. Transmission by bites of several species of ticks has also been documented [20–22]. Unlike in sub-Saharan Africa, where ASFV is maintained in a domestic and extensive sylvatic transmission cycle that involves several wild animal species, in Lithuania as well as in remaining parts of Eastern Europe ASF virus circulates in domestic pigs and European wild boars only [23].

The role of wild boars in the appearance of ASF in 2014 can be assumed as essential to the virus spread. Spillover of the disease in Lithuania is linked with infected wild boar movement from endemic zone (Belarus) [24], while continuous persistence of the virus in wild boars is linked to close contact with the carcasses of other infected wild boar [12].

In addition, ASFV strains circulating in Eastern Europe are considered to be highly virulent and induce an acute form of the disease [25]. However, experimental studies suggest that certain proportion of animals can remain asymptomatic or recover from the disease thereby becoming virus carriers and may contribute to the further spreading of the disease [9, 26, 27].

Over the past four years since the virus was introduced in Lithuania in January 2014, increased numbers of outbreaks have been reported, including two industrial scale farms, leading to incineration of approximately 19.000 and 25.000 pigs in 2014 and 2017 respectively.

Recent publication about epidemiological situation of ASF in two areas in Estonia showed a temporal and spatial differences throughout the course of the epidemic, suggesting that the first introduction of ASF may have happened several months before Estonia was officially declared as affected by ASF [28]. Low prevalence and slow spread of ASFV in backyard pig holdings in 2014 was linked to wild boar infection in Latvia [12]. Similar slow spread of the disease in areas of dense wild boar populations and a primary role of wild boars in virus maintenance and repeated introductions of ASFV were published in Poland [29]. Epidemiological situation of ASF in a population of wild boars in eastern Poland in 2014–2015 was described by Wozniakowski et al. [9]. Each ASF affected country has different prevalence in pigs and wild boars due to differences in pig production sites, wild boar density and dispersion. However, existing ASF infection studies in Baltic countries and Poland are of relatively small scale. Despite the high incidence of ASF in Lithuania and several large scale outbreaks in industrial pig farms, no scientific overview of the situation in the county has yet been published. Therefore, the aim of this study was to investigate the current status of ASF infection in populations of pig and wild boar and to fill data gaps of ASF virus prevalence in Lithuania 2014–2017.

## Materials and methods

### Surveillance program and sample collection

A National surveillance program was introduced in Lithuania after detection of the first cases of ASF in wild boars in January 2014. Due to the evolution of epidemiological situation, affected territories have been periodically revised and outlined in relevant Commission Implementing Decisions [30]. Restriction zones were established in areas with proceeding levels of risk designated in the Annex to Decision 2014/709/EU [31] that includes Part I (when a certain level of risk exists due to proximity to the infection), Part II (infection occurring only in the feral pig population) and Part III (infection in both pigs holdings and the feral pig population).

Restriction zones were established in the affected area based on the Annex to Decision 2014/709/EU that includes Part I (wild boar at risk, but no cases found risk areas with no cases and outbreaks adjacent to Part II or Part III), Part II (wild boar testing positive but no spill over into domestic pigs areas where ASF occurred in wild boars) and Part III (cases in wild boar and occasional spill over into domestic pigs areas where ASF occurred in wild boars and domestic pigs).

Surveillance program was carried out in all ASF affected territories, which consisted of passive (sample collection from wild boar carcasses) and active (sample collection from hunted wild boars surveillance). Sample collection was carried out as described in Commission Decision 2003/422/EC [32].

Throughout 2014–2017 period a total of 91,511 samples (of blood, internal organs and bone marrow*)* from hunted Wild boar (*Sus scrofa)* and 3685 samples from Wild boar carcasses were collected and tested, corresponding to 63,634 individual animals. Likewise, 80,687 organ and blood samples were tested from 72,562 domestic pigs. Samples were investigated for ASF in the National Food and Veterinary Risk Assessment Institute of Lithuania.

Sections of organ tissues were processed as 10 (*w*/*v*) homogenates in phosphate-buffered saline (PBS) and then used for DNA extraction. Blood clots from hunted wild boar and pigs were used to obtain serum samples, which were examined by ELISA.

### Antibody detection

Anti-ASFV antibodies were detected using commercial blocking (Ingezim Compac 1.1. PPA K3, Spain) ELISA kit, based on the use of monoclonal antibody against the P72 ASFV protein and ID Screen® ASF Indirect (IDvet, Grabels, France) multi-antigen (P32, P62 and P72) ELISA kit. All procedures were carried out according to manufacturer’s instructions. Test results were interpreted using spectrophotometry reading at 450 nm wavelength.The commercial Ingezim assay was assumed to be valid if the optical density (OD) ratio of the negative control (NC) to the positive control (PC) was equal to or greater than 4. The positive cut-off was calculated as NC - (NC - PC) × 0.5, while the negative cut off was calculated as NC - (NC - PC) × 0.4. The serum samples were considered positive if the average of their OD values was lower than the positive cutoff. Serum samples were considered negative if the average OD was higher than the negative cutoff. Sera that were considered doubtful had an average OD between the calculated positive and negative cutoff values.

The commercial IDvet assay was assumed to be valid if the OD of PC was greater than 0.350 and the ratio of the mean values of the PC and NC was greater than 3. For each sample, were calculated the S/PC percentage. For sera testing, samples of S/PC % less or equal to 30% were considered negative, if S/PC % was greater than 30% and less than 40% were considered doubtful and samples with S/PC % greater or equal to 40% were considered positive. In the case of positive or an inconclusive ELISA result, the sample was re-tested for antibody confirmation by indirect immunoperoxidase technique (IPT) according to the standardized operating procedure described by the EURL for ASF (CISA-INIA, Valdeolmos, Spain).

### DNA extraction and PCR analysis

Total DNA extraction was performed using DNeasy Blood & Tissue Kit (Qiagen, Germany) for serum and tissue samples, from 30 mg tissue homogenate or 300 mL serum samples according to manufacturer’s instructions. Primers F 5′ – CTG CTC ATG GTA TCA ATC TTA TCG A – 3′ and Kings‘s R 5′ – AGT ACC ACA AGA TCR GCC GT – 3′ and ASF probe 250 5′ –FAM– CCA CGG GAG GAA TAC CAA CCC AGT G – TAMRA – 3′ were used, targeting a conserved region in 3′-end of the VP72 gene [33]. Real-time PCR analysis was carried out using Luminaris Probe High ROX qPCR Master Mix. Samples were amplified using the following conditions: 2 min at 50 °C, 10 min at 95 °C, followed by 40 cycles at 95 ^o^ C for 15 s and at 60 ^o^ C for 1 min.

### Statistical analysis

Prevalence was calculated for both anti-ASF antibodies and presence of viral DNA. Annual prevalence was calculated separately for pigs and wild boars with CI = 95%. Regional, seasonal prevalence and prevalence of passive and active surveillances were also calculated. Correlations between animal densities in affected regions and recorded ASF-positive cases were calculated using Spearman’s rank correlation coefficient (with critical significance level 0.05). Statistical differences between seasonal and regional ASF-positive cases were calculated using Chi-squared test.

## Results

**Fig. 1:**
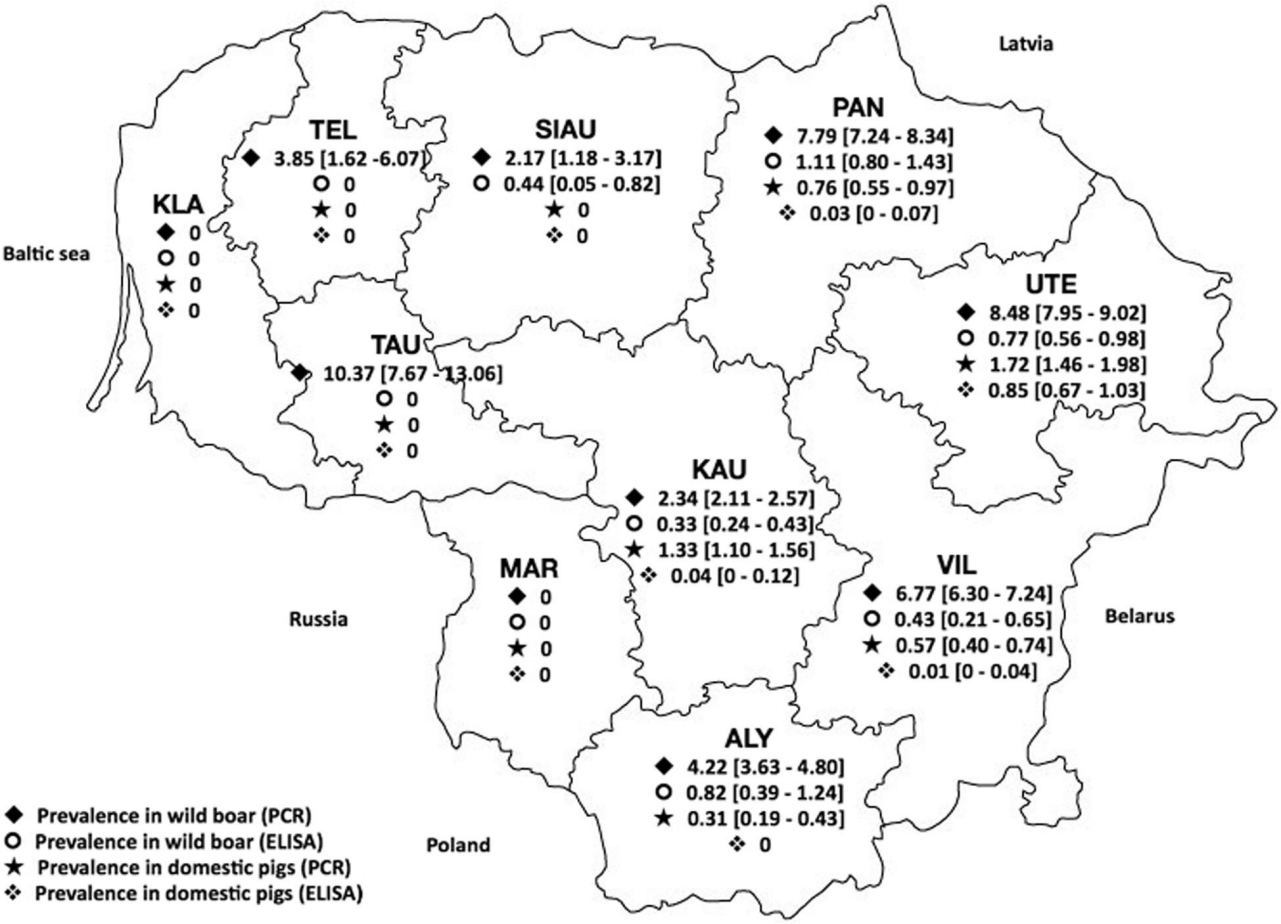
ASFV prevalence distribution in different Lithuanian counties. *Bold letters* indicate counties: *ALY* Alytus, *MAR* Marijampole, *VIL* Vilnius, *KAU* Kaunas, *TAU* Taurage, *KLA* Kaipeda, *TEL* Telsiai, *SIAU* Siauliai, *PAN* Panevezys, *UTE* Utena. *Percentage lines* indicates prevalence rate determined by the ELISA and PCR

The average prevalence of ASF-specific antibodies in wild boar population during the 2014–2017 period was 0.45% (95% CI 0.39–0.51%) based on ELISA test results (Table 1). The average prevalence of ASF virus in wild boars during the same period was 4.69% (95% CI 4.53–4.86%) based on PCR test results. Prevalence of ASF virus in wild boars gradually increased every year since the beginning of the study from 0.83% (95% CI 0.69–0.98%) in 2014 to 12.39% (95% CI 11.91–12.86%) in 2017. The average seasonal prevalence of ASF virus was statistically significant (*p* < 0.05) and ranged from 2.12% (95% CI 1.88–2.36%) in spring to 7.45% (95% CI 7.03–7.88%) in autumn. Prevalence of ASF-specific antibodies between seasons was not statistically significant. Average regional prevalence ranged from 0 to 1.11% (95% CI 0.80–1.43%) (Panevėžio county) and from 0 to 10.37% (95% CI 7.67–13.06%) (Tauragės county) for ASF specific antibodies and ASF virus respectively and were statistically significant (*p* < 0.05). Two counties were free from ASF virus and specific antibodies (Klaipėdos and Marijampolės counties) (Fig. 1).

**Table 1:** Data from ELISA and PCR assays of wild boar samples obtained from 2014 to 2017

Variable	No. positive/total (ELISA)	ELISA (%) [CI 95%]	No. positive/total (PCR)	PCR (%) [CI 95%]
Year				
2014	2/7932	0.03 [0–0.06]	122/14658	0.83 [0.69–0.98]
2015	34/7099	0.48 [0.32–0.64]	146/12527	1.17 [0.98–1.35]
2016	62/14582	0.43 [0.32–0.53]	402/17743	2.27 [2.05–2.48]
2017	98/13821	0.71 [0.57–0.85]	2317/18706	12.39 [11.91–12.86]
Season				
Winter	65/10969	0.87 [0.69–1.04]	856/21922	3.90 [3.65–4.16]
Spring	8/10141	0.08 [0.02–0.13]	291/13745	2.12 [1.88–2.36]
Summer	33/10756	0.31 [0.20–0.41]	734/13126	5.59 [5.20–5.99]
Autumn	90/11568	0.78 [0.62–0.94]	1106/14841	7.45 [7.03–7.88]
Surveillance type				
Passive	–	–	1957/2980	65.67 [63.97–67.38]
Active	196/43434	0.45 [0.39–0.51]	1030/60654	1.67 [1.57–1.78]
Wild boar total	196/43434	0.45 [0.39–0.51]	2987/63634	4.69 [4.53–4.86]

The average ASF virus prevalence in the boar carcasses (passive surveillence) was 65.67% (95% CI 63.97–67.38%) compared to hunted boars (active surveillance) where average ASF virus and specific antibody prevalence was 1.67% (95% CI 1.57–1.78%) and 0.45% (95% CI 0.39–0.51%) respectively. Yearly prevalence of passive surveillance was consistent with total yearly prevalence and a gradual increase from 20.10% (95% CI 14.46–25.74%) in 2014 to 79.68% (95% CI 77.89–81.47%) in 2017 was observed (Table 2). Seasonal prevalence of passive surveillance was not consistent with total seasonal prevalence and ranged from 61.00% (95% CI 57.22–64.77%) in autumn to 83.23% (95% CI 80.30–86.15%) in winter.

**Table 2:** Prevalence of ASFV infection in wild boars carcasses

Variable	No. positive/total (PCR)	PCR (%) [CI 95%]
Year		
2014	39/194	20.10 [14.46–25.74]
2015	43/186	23.12 [17.06–29.18]
2016	344/656	52.44 [48.62–56.26]
2017	1549/1944	79.68 [77.89–81.47]
Season		
Winter	521/626	83.23 [80.30–86.15]
Spring	564/909	62.05 [58.89–65.20]
Summer	499/804	62.06 [58.71–65.42]
Autumn	391/641	61.00 [57.22–64.77]
Passive surveillance total	1957/2980	65.67 [63.97–67.38]

The average prevalence of ASF-specific antibodies and virus in pigs during years 2014–2017 was 0.12% (95% CI 0.10–0.15%) and 0.86% (95% CI 0.78–0.95%) respectively (Table 3). Prevalence of ASF virus in pigs ranged from 0.24% (95% CI 0.17–0.32%) in 2015 to 2.74% (95% CI 2.33–3.15%) in 2017. Seasonal prevalence of ASF virus ranged from 0% in spring to 3.68% (95% CI 3.32–4.05%) in summer and differences between seasonal prevalence were statistically significant (*p* < 0.05). Regional prevalence ranged from 0 to 0.85% (95% CI 0.67–1.03%) (Utenos county) and from 0 to 1.72% (95% CI 1.46–1.98%) (Utenos county) for ASF specific antibodies and virus respectively and the differences between regional prevalence were statistically significant (*p* < 0.05). Five counties were free from ASF virus (Klaipėdos, Marijampolės, Šiaulių, Tauragės and Telšių counties) and six counties were free from ASF specific antibodies (Alytaus, Klaipėdos, Marijampolės, Šiaulių, Tauragės and Telšių counties) (Fig. 1).

**Table 3:** Data from ELISA and PCR assays of domestic pigs samples obtained from 2014 to 2017

Variable	No. positive/total (ELISA)	ELISA (%) [CI 95%]	No. positive/total (PCR)	PCR (%) [CI 95%]
Year				
2014	83/24033	0.35 [0.27–0.42]	148/19657	0.75 [0.63–0.87]
2015	0/22826	0	38/15586	0.24 [0.17–0.32]
2016	3/13651	0.02 [0–0.05]	58/6121	0.95 [0.7–1.19]
2017	4/12052	0.03 [0–0.07]	164/5985	2.74 [2.33–3.15]
Season				
Winter	0/19045	0	4/9817	0.04 [0–0.8]
Spring	0/16543	0	0/13704	0
Summer	90/15427	0.58 [0.46–0.70]	376/10216	3.68 [3.32–4.05]
Autumn	0/21547	0	28/13612	0.21 [0.13–0.28]
Domestic pigs total	90/72562	0.12 [0.10–0.15]	408/47349	0.86 [0.78–0.95]

A correlation between the pig density and number of recorded pig ASF-positive cases in affected regions was found in 2017 (correlation coefficient *R* = 0.78, *p* < 0.05) but did not extend to the earlier years of the study where correlation was no longer statistically significant. No correlation was detected between the wild boar density and number of recorded pig or wild boar ASF-positive cases.

## Discussion

On 14 January 2014, Lithuania’s National Food and Veterinary Risk Assessment Institute confirmed two ASF cases in wild boars in area bordering with Belarus (VIL, Fig. 1). Since then, the country underwent a rapid spread of ASF almost simultaneously with three other EU countries: Poland, Latvia and Estonia. To date, more than three thousand of ASF notifications in wild boars have been reported in Lithuania alone [34]. The average prevalence of ASFV in wild boars during the 2014–2017 period was 4.69% which greatly outnumbers outbreaks among domestic pigs, where average viral prevalence was 0.86%. Despite the fact, that the number of outbreaks in pig farms was several times smaller compared to the incidence rate in wild boars, ASF is a major threat to local pig industry today. ASFV mostly affects backyard holdings that gradually ruin small-scale farming.

Although a correlation between the pig density and number of recorded pig ASF- positive cases in affected regions of Lithuania was found in 2017 (*R* = 0.78, *p* < 0.05) it did not extend to the earlier years of the study where correlation was no longer statistically significant. This can be explained by a small number of infected areas where ASF cases had only recently been detected. The analysis of combined data from Poland and the other Baltic States, conducted by a panel of European Food Safety Authority experts, found no correlation between wild boar density and ASF case notifications [24]. However, this uncertainty probably occurs due to inaccurate data on the number of wild boars since European Union countries are lacking of standardized accounting method for wild animals. Overall, it might be concluded that population density in wild boars does not correlate with virus prevalence in a given region, but might influence the risk of virus introduction to a new wild boar group due to increase in adult male dispersal distance. In turn, maintaining a low wild boar population levels might prevent long range dispersals of adult males. In addition, low-density barrier strategy in the surrounding areas has been previously proposed [29] although the effectiveness of the said strategy is debatable since the lack of possible mates for migrating male boars might increase their dispersal range even more.

Our data shows that specific anti-ASFV antibodies in wild boars tested in the framework of active surveillance appear at a very low rate. Based on ELISA test results in Lithuanian wild boar serum samples, the average prevalence of ASF specific antibodies during 2014–2017 was only 0.45%. However, it indicates that some wild boars can survive early stages of ASF infection and transmit the virus. This finding is confirmed by experimental infection of pigs with Lithuanian wild boar isolate LT14/1490 that is responsible for the first ASF case in Lithuania. ASF specific antibodies were detected 17–18 days after inoculation in 33% of experimentally infected pigs, while 10% of them survived the infection showing weak and intermittent peaks of viraemia [26].

ASF viral DNA prevalence in hunted wild boars using PCR analysis gradually increased from 0.83 to 2.27% in 2014 to 2016 respectively. However, 2017 saw a dramatic jump in the number of ASF positive wild boar cases resulting in prevalence of 12.39%. More than fivefold increase in ASF positive wild boar cases in 2017 compared to 2016 could be explained by extensive spatial spread of ASF infection in Lithuanian wild boar population during this period, since before 2016, ASF virus in Lithuania was restricted to a limited geographical area. In addition, intense active surveillance program in 2017, emergence of ASF in naïve wild boars in previously ASF-free regions and high level of wild boar density could have contributed to elevated prevalence. Passive surveillance showed a similar increase of ASF positive cases in 2017. Process of carcass collection has been actively and successfully encouraged in Lithuania by monetary incentives for each wild boar carcass retrieved, explaining overall ASFV prevalence rate jump in 2017.

Study results show seasonal prevalence of ASFV infection in wild boar population. We found statistically significant difference (*p* < 0.05) between the number of ASFV cases in winter and summer or autumn with prevalence rates of 3.90, 5.59 and 7.45% respectively. Differences in ASFV prevalence in wild boars could be explained by a more effective retrieval of carcasses in winter as part of passive surveillance program due to easier visualization of the carcass against a snowy background and lack of vegetation that may obstruct the view in other seasons. Similar statistically significant (*p* < 0.05) seasonal distribution was obtained by testing specific anti-ASF antibodies.

ASFV infection in Lithuanian wild boar population spread gradually across most of the country resulting in different prevalence rates with eight out of ten Lithuanian counties being affected. Statistically significant (p < 0.05) spatial differences could be explained by wild boar concentration differences in affected regions and temporal distribution factors.

Results of passive surveillance and total ASF positive cases did not show significant seasonal difference. ASF infected wild boar carcasses were found at very similar 61.00–62.06% prevalence rates in spring, summer or autumn, however highest level of ASF positive carcasses were found in winter.

The average prevalence of ASF-specific antibodies in 2014–2017 in Lithuanian pigs was only 0.35%, while during 2015–2017 period ASF-specific antibodies were detected in only 0.02–0.03% of tested serum samples (*n* = 48,530). A relatively low antibody prevalence could be due to the fact that most of ASF infected pigs are diagnosed and sampled in early stages of the disease, while stable specific antibody count is usually achieved at least two weeks after the infection [26].

The average ASF virus prevalence using PCR method in 2014–2017 was 0.86%, however a jump in positive cases, similar to wild boars, was observed in 2017 with 2.74% of tested pigs being ASF positive. ASFV infection in Lithuanian pig population spread gradually across most of the country, mainly in backyard holdings with low levels of biosecurity. All counties with positive pig cases overlapped with wild boar positive counties, indicating that regional ASF prevalence in wild boars is a risk factor for domestic pig farms in that particular region, especially where direct contact between wild boar and pig populations might be possible (e.g. backyard holdings).

Seasonal distribution of ASF cases in pigs showed summer to be a statistically significant (*p* < 0.05) factor for ASF outbreaks. All outbreaks in back yard holdings and two commercial farms in Lithuania during 2014–2017 have been detected in summer - mainly end of July and August. ASF outbreak peaks in summer could be attributed to human or insect activity with capabilities of virus transfer which could play a role in epidemiology of ASFV in pig population. A reason for dramatic increase of ASF cases in Lithuanian pigs during July and August needs to be further investigated. It would be useful to test if such outbreak tendencies could be observed in Poland, Latvia and Estonian pig population.

The ASF virus is extremely resistant to environment factors. It can persist in relatively high temperatures and processes of putrefaction. These properties provide excellent conditions for mushroom hunters to mechanically transport the virus. Therefore, the main challenge seems to be not the infected wild boars that are prone to contact with domestic pigs in small holdings, but high tenacity of the virus and its ability to be transmitted by indirect contact. Recent experimental studies indicated that domestic pig contact with infected wild boars is sufficient to transmit the infection [25, 35, 36] and there are no reliable evidence supporting the assumption that the spread of the disease in Lithuania is associated with direct contact between two mentioned species.

## Conclusions

The primary forecasts after virus introduction to Lithuania had predicted that virus would quickly spread throughout the country and destroy entire pig industry. However, this predicted pessimistic scenario did not occur. It may be due to local landscape and timely used methods for control (fences preventing movement to non-effected areas, female-targeted boar hunting, carcass removing). On the other hand, in 2017, the number of ASF positive cases in both domestic pigs and wild boars had increased significantly, therefore control measures should be applied and iterated continuously, including feeding bans, reduction of the domestic pig population in the backyard holdings and improvement of biosecurity regulations. Continuous ASF virus surveillance program is also needed.

## Abbreviations

ASF: African Swine Fever
ASFV: African swine fever virus
CI: confidence interval
ELISA: Enzyme-Linked ImmunoSorbent Assay
NC: Negative control
OD: optical density
PC: positive control
PCR: polymerase chain reaction
